# High anion gap metabolic acidosis caused by D-lactate: mind the time of blood collection

**DOI:** 10.11613/BM.2020.011001

**Published:** 2019-12-15

**Authors:** Matthias Weemaes, Martin Hiele, Pieter Vermeersch

**Affiliations:** 1 Clinical Department of Laboratory Medicine, UZ Leuven, Leuven, Belgium; 2 Clinical Department of Gastroenterology, UZ Leuven, Leuven, Belgium; 3 Department of Cardiovascular Sciences, University of Leuven, Leuven, Belgium

**Keywords:** D-lactate, D-lactic acidosis, high anion gap acidosis, preanalytical phase, case report

## Abstract

**Introduction:**

D-lactic acidosis is an uncommon cause of high anion gap acidosis.

**Materials and methods:**

A 35-year old woman was admitted to the emergency room with somnolence, drowsiness, dizziness, incoherent speech and drunk appearance. Her past medical history included a Roux-en-Y bypass. Point-of-care venous blood analysis revealed a high anion gap acidosis. Based on the clinical presentation, routine laboratory results and negative toxicology screening, D-lactate and 5-oxoprolinuria were considered as the most likely causes of the high anion gap acidosis. Urine organic acid analysis revealed increased lactate, but no 5-oxoproline. Plasma D-lactate was < 1.0 mmol/L and could not confirm D-lactic acidosis.

**What happened:**

Further investigation revealed that the blood sample for D-lactate was drawn 12 hours after admission, which might explain the false-negative result. Data regarding the half-life of D-lactate are, however, scarce. During a second admission, one month later, D-lactic acidosis could be confirmed with an anion gap of 40.7 mmol/L and a D-lactate of 21.0 mmol/L measured in a sample collected at the time of admission.

**Main lesson:**

The time of blood collection is of utmost importance to establish the diagnosis of D-lactic acidosis due to the fast clearance of D-lactate in the human body.

## Introduction

Humans lack a D-lactate dehydrogenase and the lactic acid pool (1-2 mmol/L) normally consists exclusively of L-lactate, although traces of D-lactate might be formed by the glyoxylate pathway from methylglyoxal (*1*-*4*). Humans can also take up D-lactate *via* the gastrointestinal tract from fermented food products (*e.g.* yogurt) or produced by colonic bacterial flora from undigested simple carbohydrates (*3*-*5*). Under normal circumstances, most simple carbohydrates are reabsorbed in the small intestine and only small amounts of L- and D-lactate are produced in the colon. In patients with short bowel syndrome, however, larger amounts of simple carbohydrates can reach the colon after a carbohydrate rich meal, which can result in significant D-lactate production (via bacterial D-lactate dehydrogenase or LD-lactate racemase) and subsequent uptake via the gastrointestinal tract (*3*, *5*, *6*). Patients with bacterial overgrowth of the small intestine are also at risk of increased D-lactate uptake.

D-lactic acidosis is considered one of the most enigmatic causes of high anion gap acidosis because routine enzymatic measurement of lactate in blood only measures L-lactate due to the substrate specificity of L-lactate dehydrogenase and L-lactate oxidase. These enzymes have no affinity for L-lactate’s mirror image and enantiomer D-lactate. Atypical neurological symptoms accompanied by elevated serum anion gap acidosis should warrant thorough investigation and exclusion of a possible D-lactic acidosis if other causes of high anion gap acidosis remain negative.

## Case description

A 35-year old woman was admitted to the emergency room with drowsiness, dizziness, incoherent speech, drunk appearance, and alternating episodes of somnolence and agitation/aggression. Her mother, who accompanied her, told she had had several similar episodes during the last 2 years, but none as severe as the current one. Her past medical history included osteomalacia, a Roux-en-Y bypass following a complicated cholecystectomy, and a vena cava filter for deep venous thrombosis and subsequent lung embolism. Her blood pressure was 94/65 mmHg, heart rate was 83/min and her body temperature was normal. Neurological examination revealed an atactic finger-nose test and heel-shin test, and a bilateral adduction paresis of the eyes.

## Laboratory analyses and other diagnostic evaluations

Laboratory tests at the time of admission showed a high anion gap acidosis (Table 1). No abnormalities were found on CT brain (without intravenous contrast). There was no evidence for ingestion of drugs, methanol or glycols. Urine dipstick analysis and urine toxicology screening did not reveal any abnormalities.

**Table 1:** Laboratory findings at first admission

**Parameter (unit)**	**Result**	**Reference interval**
**Venous blood gas sample (ABL90 Flex)**		
pH (pH units)	7.24	7.35 – 7.43
pCO_2_ (kPa)	3.5	4.7 – 6.0
pO_2_ (kPa)	4.8	11.3 – 13.8
HCO_3_^-^ (mmol/L)	11	22 – 29
Base excess (mmol/L)	- 15	- 2.0 to + 3.0
Haemoglobin (g/L)	133	120 – 160
Haematocrit (L/L)	0.408	0.370 – 0.470
Sodium (mmol/L)	146	136 – 146
Potassium (mmol/L)	4.0	3.5 – 4.5
Chloride (mmol/L)	114	98 – 106
Anion gap (mmol/L)	25.0	10.0 – 20.0
Glucose (mmol/L)	4.5	3.9 – 5.8
Lactate (mmol/L)	1.2	0.5 – 2.2
**Venous blood sample (Roche Cobas 8000)**		
Urea (mmol/L)	1.7	≤ 8.2
Creatinine (µmol/L)	98.1	45.1– 84.0
eGFR (CKD-EPI) (mL/min/1.73 m^2^)	64	≥ 90
Calcium (mmol/L)	2.23	2.15 – 2.55
Phosphate (mmol/L)	1.29	0.81 – 1.45
Total protein (g/L)	59	66-88
Albumin (g/L)	37	35 – 52
CRP (mg/L)	0.5	≤ 5.0
AST (U/L)	56	≤ 31
ALT (U/L)	92	≤ 31
GGT (U/L)	19	≤ 40
Bilirubin total (µmol/L)	7.9	≤ 20
Ethanol (g/L)	< 0.1	< 0.1
**Urine**		
Toxicology screening	Negative	Included: amphetamines, antidepressants, barbiturates, benzodiazepines, cannabinoids, cocaine and metabolites, opioids, synthetic opioids, phenothiazines, antipsychotics, paracetamol and salicylates.
Dipstick	Negative	Included: leukocyte esterase, nitrite, protein, glucose, ketones, haemoglobin, urobilinogen, bilirubin, pH 5.0

## Considered diagnoses and further investigations

Wernicke’s encephalopathy was considered in the differential diagnosis for the neurologic abnormalities, but could not account for the metabolic acidosis. The high anion gap acidosis at admission pointed towards the presence of an unmeasured anion. A number of mnemonics have been proposed over the years to assess the cause of high anion gap metabolic acidosis, including KUSMALE (*i.e.* ketoacidosis, uraemia, salicylate poisoning, methanol, aldehyde (paraldehyde), lactate, and ethylene glycol), CATMUDPILES (*i.e.* carbon monoxide, cyanide, congenital heart failure, aminoglycosides, theophylline, toluene, methanol, uraemia, diabetic ketoacidosis, paraldehyde, iron, isoniazid, lactate, ethanol, ethylene glycol, diethylene glycol, or propylene glycol, salicylates) and GOLDMARK (*i.e.* glycols, 5-oxoproline (linked to paracetamol use), L-lactate, D-lactate, methanol, aspirin, renal failure and ketoacidosis). The acronym GOLDMARK, which was proposed in 2008, is the most frequently used (*7*).

Based on the clinical presentation and initial laboratory results at time of admission and using the GOLDMARK-acronym, 5-oxoproline and D-lactate were considered as potential causes of the raised anion gap metabolic acidosis. The treating physician ruled out renal failure as a potential cause since plasma creatinine was only slightly elevated.

Urinary organic acid analysis was performed to rule out 5-oxoprolinuria and a sample was sent to the laboratory for the quantification of D-lactate in plasma to rule out D-lactic acidosis. Urinary organic acid analysis revealed no 5-oxoprolin, but an elevated urinary lactate (3454 mmol/mol creatinine, ≤ 46 mmol/mol creatinine). Plasma D-lactate was measured using an in-house assay with D-lactate specific lactate dehydrogenase (D-LD, from *Lactobacillus leichmannii*) at 340 nm (*8*). D-lactate is calculated by monitoring kinetically the conversion of NAD^+^ to NADH at 340 nm in the presence of D-LD. Before the D-LD is added, alanine aminotransferase and glutamate are added to deplete any available pyruvate in the sample. The conversion of D-lactate to D-pyruvate is promoted by performing the reaction at an alkaline pH (9.5) and by adding excess NAD^+^. D-lactic acid was, however, only slightly elevated (0.6 mmol/L, reference interval ≤ 0.2 mmol/L) and could not confirm D-lactate as the cause of the high anion gap acidosis. Further investigation revealed the blood sample for D-lactate measurement was drawn the next morning, twelve hours after admission, potentially explaining the only slight increase in D-lactate in plasma. Given the clinical suspicion, the patient was put on a strict carbohydrate-poor diet, and given neomycine antibiotic treatment and adequate rehydration (with supplementation of HCO_3_^-^). The patient recovered and was discharged from the hospital with a tentative diagnosis of D-lactic acidosis due to bacterial overgrowth in a patient with short bowel following a Roux-en-Y bypass.

One month later, the patient was readmitted with the same symptoms. Laboratory results at the time of admission revealed a high anion gap (40.7 mmol/L, reference interval 9.0 - 20.0) acidosis, normal osmolality (295 mOsm/kg, reference interval 275 - 295 mOsm/kg) and markedly elevated D-lactate (21.0 mmol/L), confirming D-lactic acidosis as the cause of the high anion gap acidosis.

## What happened?

The fact that the diagnosis could not be confirmed during the first episode was because D-lactate had been measured in a sample drawn 12 hours after admission. In hindsight, the false-negative result can be explained by the short half-life of D-lactate. Data regarding the half-life of D-lactate are, however, scarce. De Vrese *et al.* reported a half-life of D-lactate of 28.6 ± 4.3 and 40.4 ± 5.4 minutes with peak plasma concentrations of 0.4 and 0.5 mmol/L, respectively, in healthy volunteers following oral administration of a racemic DL-lactate mixture (*9*). The elevated lactate in urine measured with gas chromatography–mass spectrometry (GC-MS), with normal L-lactate in plasma measured on Cobas (Roche diagnostics, Rotkreuz, Switzerland) supports the suspected diagnosis of D-lactic acidosis since the GC-MS method does not distinguish between the stereoisomers L- and D-lactate.

During the second visit to the emergency department one month later, D-lactate was measured at the time of admission in the same sample as the anion gap and the osmolality. The increase of the anion gap above the upper limit of normal of 20 mmol/L (40.7 mmol/L - 20 mmol/L = 20.7 mmol/L) matched the plasma D-lactate concentration (21.0 mmol/L), confirming the diagnosis of D-lactic acidosis.

## Discussion

In a patient with rapidly developing neurological symptoms combined with an unexplained raised anion gap acidosis, physicians should always be mindful of possible D-lactic acidosis. There are four possible sources of D-lactate in the human body. The first is the methylglyoxal pathway, which produces D-lactate in only small quantities (*1*). Other sources of D-lactic acidosis include fermented foods (*e.g.* yoghurt, sour cabbage and pickles), peritoneal dialysate and Ringer lactate (both are racemic mixtures of L- and D-lactate) (*6*). Propylene glycol, sometimes contained in intravenous medication used in intensive care units or used as an autointoxication means, can be converted to D-lactate via both above mentioned pathways (*10*).

The most important source of D-lactate in patients with D-lactic acidosis is the colonic bacterial flora. In persons with short bowel syndrome, ingested sugars are not fully absorbed by the small intestine but are fermented by colonic bacteria to L- or D-lactate, depending on the composition of the colonic microbiome. The fermentation process causes the pH to drop to a level where acid-resistant D- and L-lactate producing bacteria outgrow other non-lactate producing bacteria (*4*). The massively produced D-lactate is absorbed by the intestinal mucosa. Besides short bowel syndrome, other risk factors for D-lactic acidosis include decreased intestinal motility and treatment with antibiotics (*e.g.* tetracycline, metronidazole, trimethoprim-sulfamethoxazole, doxycycline and vancomycin) which can trigger intestinal bacterial overgrowth (*6*). Lactulose therapy and the use of probiotics have also been reported as causes of D-lactic acidosis (*11*, *12*).

Although humans lack D-lactate dehydrogenase, D-lactate can be metabolized to pyruvate by the intramitochondrial enzyme D-2-hydroxyacid dehydrogenase (D-2-HDH) (*13*). Metabolization of D-lactate via D-HDH is considered the main mechanism of D-lactate clearance but D-lactate is also cleared via the kidneys (*2*). The renal threshold for D-lactate is much lower than for L-lactate with less than 50% reabsorption for plasma concentrations of 4 - 6 mmol/L (*14*). Renal reabsorption of D-lactate is also hindered in the presence of L-lactate, suggesting that both enantiomers use the same renal transporter for reabsorption (*2*). While the renal clearance of D-lactate is < 2% for plasma concentrations of < 0.5 mmol/L, a renal clearance of > 30% has been observed for higher D-lactate concentrations (*9*). The increased urinary lactate on organic acid analysis in our patient confirms that there is significant renal clearance of D-lactate in case of D-lactic acidosis.

Data on plasma half-life of D-lactate scarce. De Vrese *et al.* reported a half-life of D-lactate of 28.6 ± 4.3 and 40.4 ± 5.4 minutes with peak plasma concentrations of 0.35 and 0.5 mmol/L, respectively, in healthy volunteers. It should be noted that this study, like all other studies examining D-lactate metabolism in healthy volunteers, was performed with a racemic mixture, which might have impacted the results. Moreover, the peak plasma D-lactate concentrations in this study and other studies examining D-lactate metabolism in healthy volunteers were significantly lower than in our patient (≤ 5.7 mmol/L) (*2*, *14*).

## What you can do in your laboratory to prevent such errors

1. D-lactic acidosis should be considered in the differential diagnosis in patients presenting with atypical neurological presentation and high anion gap acidosis (GOLDMARK).
2. D-lactate should be measured in a sample collected as soon as possible after the onset of symptoms due to the short half-life of D-lactate.
3. Confirming D-lactic acidosis can, however, be challenging since the symptoms can rapidly appear and resolve (*e.g.* after a carbohydrate rich meal) and because of the relatively short half-life of D-lactate. Retesting during a subsequent episode might be necessary to confirm the diagnosis.
